# *De Novo* Transcriptome Analysis Reveals Potential Thermal Adaptation Mechanisms in the Cicada *Hyalessa fuscata*

**DOI:** 10.3390/ani11102785

**Published:** 2021-09-24

**Authors:** Hoa Quynh Nguyen, Yuseob Kim, Yikweon Jang

**Affiliations:** 1 Interdisciplinary Program of EcoCreative, Ewha Womans University, Ewhayeodaegil-52, Seoul 03760, Korea; hoanq8x@gmail.com (H.Q.N.); yuseob@ewha.ac.kr (Y.K.); 2 Department of Life Sciences and Division of Ecoscience, Ewha Womans University, Ewhayeodaegil-52, Seoul 03760, Korea; 3 Institute of Chemistry, Vietnam Academy of Science and Technology, No. 18 Hoang Quoc Viet Street, Cau Giay District, Hanoi 10072, Vietnam

**Keywords:** *Hyalessa fuscata*, thermal tolerance, transcriptomes, heat shock proteins, antioxidants, energy metabolism

## Abstract

**Simple Summary:**

In metropolitan Seoul and its vicinity, cicadas of the species *Hyalessa fuscata* living in warmer areas could tolerate the heat better than those living in cooler areas, but genetic mechanisms involved in better heat tolerance remained unclear. In this study, we examined differences in gene expression of cicadas living in a warm urban area, a cool urban area and a suburban area in three experimental treatments: no heating, 10 min heating and heating until the cicadas lost their mobility. Cicadas from the warm urban area changed their gene expressions the most. Activated genes were mostly related to heat shock, energy metabolism, and detoxification. These results suggested that under heat stress, cicadas inhabiting warm areas could differentially express genes to increase their thermal tolerance.

**Abstract:**

In metropolitan Seoul, populations of the cicada *Hyalessa fuscata* in hotter urban heat islands (“high UHIs”) exhibit higher thermal tolerance than those in cooler UHIs (“low UHIs”). We hypothesized that heat stress may activate the expression of genes that facilitate greater thermal tolerance in high-UHI cicadas than in those from cooler areas. Differences in the transcriptomes of adult female cicadas from high-UHI, low-UHI, and suburban areas were analyzed at the unheated level, after acute heat stress, and after heat torpor. No noticeable differences in unheated gene expression patterns were observed. After 10 min of acute heat stress, however, low-UHI and suburban cicadas expressed more heat shock protein genes than high-UHI counterparts. More specifically, remarkable changes in the gene expression of cicadas across areas were observed after heat torpor stimulus, as represented by a large number of up- and downregulated genes in the heat torpor groups compared with the 10 min acute heat stress and control groups. High-UHI cicadas expressed the most differentially expressed genes, followed by the low-UHI and suburban cicadas. There was a notable increase in the expression of heat shock, metabolism, and detoxification genes; meanwhile, immune-related, signal transduction, and protein turnover genes were downregulated in high-UHI cicadas versus the other cicada groups. These results suggested that under heat stress, cicadas inhabiting high-UHIs could rapidly express genes related to heat shock, energy metabolism, and detoxification to protect cells from stress-induced damage and to increase their thermal tolerance toward heat stress. The downregulation of apoptosis mechanisms in high-UHI cicadas suggested that there was less cellular damage, which likely contributed to their high tolerance of heat stress.

## 1. Introduction

Insects are poikilotherms whose body temperature changes with the ambient temperature. Indeed, temperature affects all levels of insects’ activities from the molecular level, such as the rate of chemical reactions and metabolic processes in vivo [1]; to the organismal level, such as alternating phenological patterns [2,3] and oviposition frequency [4]; to the population level, such as shaping the distribution of the species [4]. Under certain conditions, insects employ specific behavioral and physiological mechanisms to adjust to their physical habitat conditions [5,6]. Several studies investigated the underlying adaptive mechanisms in genetically different populations of various species [7,8,9,10,11], yet none have investigated transcriptional changes in cicada populations living under diverse thermal regimes.

Upon exposure to heat stress, an instant cellular defense mechanism against potential macromolecular damage is activated to mitigate potential stress-induced injury and to facilitate the restoration of cell homeostasis [12,13,14,15]. Heat shock proteins (Hsps) are highly conserved heat response proteins in both prokaryotes and eukaryotes [1,12,15,16,17], and their role in heat tolerance was studied in detail [18,19]. Hsp families encompass both constitutive and heat-inducible members [18]. In the absence of heat stress, Hsps play a crucial role as molecular chaperones, facilitating the folding and assembly of proteins [20,21,22]. When exposed to high temperatures, Hsps protect cells and organisms from temperature impairments by counteracting the accumulation of aberrant proteins. Specifically, they mediate proper protein folding, repair denatured proteins, bind to unfolded proteins to prevent inappropriate protein–protein interactions or clustering of non-native conformations, and degrade or remove abnormal proteins from the cell [12,13,18,23,24,25,26]. As thermal stress increases, the abundance of Hsps increases [11], suggesting that Hsps enhance an organism’s thermotolerance [27,28].

Thermal stress is responsible not only for the activation of Hsps but also for the breakdown of the balance between reactive oxygen species (ROS) and antioxidants [29,30]. Surplus generation of ROS can lead to apoptosis and DNA damage [31,32]. In this scenario, antioxidant enzymes are triggered as a defensive mechanism to scavenge ROS [33], where the term “antioxidant gene” is ascribed to those genes that directly interact with reactive species detoxification [34]. The thermal tolerance of several insects was linked to the upregulation of antioxidant genes [13,35].

Furthermore, genes relating to energy metabolism also deserve careful examination. The energy-limited tolerance hypothesis argues that under stressful conditions, energy expenditure is reallocated in such a way that basal maintenance processes for survival take priority over other physiological processes [36]. The chaperone function of Hsps is ATP-dependent and usually requires high energy expenditure [37,38,39,40]. Therefore, without the assistance of genes involved in energy metabolism, Hsps might not function effectively and basal maintenance might not be sustained, which would ultimately lower insect thermal tolerance and lead to irreversible cell death [1].

During thermal stress, other mechanisms that allow for sensing and transduction of intracellular immune responses, DNA damage, and repair are also altered in insects [10,41]. The energy budget is reallocated toward maintenance over other functions, and growth and proliferation are temporarily inhibited until cell homeostasis is fully recovered or permanently suppressed when severe thermal stress causes irreversible cell recovery. A key feature of the cell stress response is the expression of intracellular immune responses to further facilitate protection from macromolecular damage [10]. In addition, signal transduction pathways related to the reallocation of energy expenditure to basal maintenance are activated [10,41]. In the event that mechanisms to secure cell survival fail, the equilibrium between net growth and net death of cells is disrupted, leading to the activation of programmed cell death to eradicate damaged cells [42].

In metropolitan Seoul, the population of *Hyalessa fuscata* (Hemiptera: Cicadidae) has been expanding rapidly in recent decades. Enumeration of cicada exuviae revealed higher population densities in warmer urban areas than cooler urban areas and the areas surrounding Seoul [43]. This was attributed to the urban heat island (UHI) effect, which is a phenomenon that occurs when urban cores become hotter than the surroundings [44]. A positive relationship between the thermal responses of cicadas and the intensity of the urban heat island effect was documented [45]. This finding suggests the potential adaptation of this cicada species to warm urban cores in Seoul.

To elucidate the role of gene expression in the thermal adaptation of *H. fuscata* to a hot urban environment, we conducted a de novo transcriptome-wide analysis of cicadas. Adults of *H. fuscata* were collected from three areas in and around Seoul representing three different temperature conditions: an urban area with a high urban heat island intensity (high-UHI area), an urban area with a low UHI intensity (low-UHI area), and an area surrounding Seoul (suburban area). We assessed the variations in gene expression profiles of *H. fuscata* individuals from these different UHI environments without being heated, after exposure to acute heat stress, and after exposure to heat torpor. We hypothesized that under exposure to heat stress, a greater extent of gene expressions that are likely involved in facilitating thermal tolerance would be observed in high-UHI cicadas than those from cooler areas. Examination of the thermal responses exhibited by *H. fuscata* from various thermal conditions revealed that the local thermal regime was strongly associated with the cicadas’ heat torpor temperatures, but not with the minimum body temperature during fully coordinated activities or the upper thermoregulatory point for shade-seeking behavior [45]. For these reasons, we predicted that: (1) no transcriptional differences under unheated conditions would be exhibited across cicadas sampled from different thermal areas; (2) no significant transcriptional changes would be observed in response to acute heat stress, where no thermoregulating behaviors were present; and (3) cicadas that were sampled from warmer urban areas would show greater transcriptome changes related to heat endurance under heat torpor than cicadas that were sampled from cooler urban areas. In particular, we hypothesized that the genes related to heat shock, energy metabolism, antioxidant defenses, detoxification, intracellular immune responses, and signal transduction pathways would be more highly expressed in cicadas from warmer urban environments than those from cooler urban environments, while genes related to programmed cell death would be expressed at lower levels in cicadas from warmer urban environments.

## 2. Materials and Methods

### 2.1. Sample Preparation and Thermal Experiments

Cicadas are subterranean as nymphs for most of their lives and only briefly become terrestrial as adults in summer. Here, only adult *H. fuscata* cicadas were chosen as study samples. Three sampling areas representing high-UHI, low-UHI, and suburban areas of metropolitan Seoul were selected: Seocho (127°1′13.7994″ E, 37°30′7.5594″ N), Nowon (127.0669° E, 37.6308° N), and Jookyo (126°50′3.84″ E, 37°39′55.08″ N), respectively [45]. From 1 June 2010 to 31 August 2016, the average maximum ambient temperatures in summer in these areas followed a descending thermal gradient, decreasing gradually from 30.97 °C in the high-UHI area to 29.02 °C in the suburban area. Ambient temperature is the sole environmental factor related to urbanization that differs between these areas [43].

To determine which molecular mechanisms are involved in the thermal adaption of cicadas, we conducted an array of thermal experiments (Supplementary Material Figure S1). First, gene expressions were compared between unheated individuals from the three areas. Second, cicadas from the three areas were exposed to an acute 10 min thermal stress, where no thermoregulating behaviors were observed in cicadas [43]. Finally, heat torpor gene expression was investigated. We expected that observing gradual transcriptional changes over the course of heat stress may allow us to distinguish changes in gene expressions invoked by heat stress exposure and, at the same time, to compare thermal responses in terms of transcriptomes between cicadas from the three areas. Cicadas were collected in two visits to each area, one in July 2017 and the other in August 2019. In 2017, gene expression profiles of heat torpor and control cicadas were investigated, and these are referred to as the heat torpor and control groups, respectively. In 2019, we studied the gene expression of cicadas that were continuously exposed to 10 min of acute heat stress, which is referred to as the 10 min group. Samples were gathered from residential complexes in the morning and experiments were executed immediately in the afternoon of the capture day. In total, nine individuals per area were captured and three individuals per group were examined.

The body temperatures of the cicadas were assessed using a 450-AKT digital thermometer with a k-type thermocouple (Omega, Stamford, CT, USA). Temperature measurements were performed without physical contact with the cicada body to prevent heat transfer. Heat torpor temperatures were determined by exposing cicadas to a heat source until no physical movement was observed [46]. Ten-minute temperatures were obtained after the cicadas were constantly heated for 10 min. We did not measure the body temperatures of the control group. Pronotum tissues of all individuals after the trial were immediately dissected and stored at −80 °C to preserve the total RNA. They were later transferred to Macrogen Inc. (Seoul, Republic of Korea) for RNA extraction and RNA-seq analysis. The total RNA integrity was checked using an Agilent Technologies 2100 Bioanalyzer (Agilent Technologies, Waldbronn, Germany) and samples with an RNA integrity number (RIN) value approximately greater than or equal to 7 were used in downstream applications.

### 2.2. TruSeq mRNA Library Construction and Illumina Sequencing

A volume of 3 µL was taken from each RNA sample as input material for cDNA library qualification. cDNA libraries were generated using the TruSeq RNA Sample Prep Kit v2 kit (Illumina, Inc., San Diego, CA, USA). Libraries were sequenced on a HiSeq4000 platform (Illumina, Inc., San Diego, CA, USA), and 100 bp/100 bp paired-end reads were generated. To verify the sizes of PCR enriched fragments, the template size distribution was checked using an Agilent Technologies 2100 Bioanalyzer (Agilent Technologies, Waldbronn, Germany) and a DNA 1000 chip.

### 2.3. De novo Transcriptome Assembly

FastQC software (Babraham Institute, Babraham, Cambridge, United Kingdom) was employed for quality control checks of the raw sequence data. Artifacts, such as low-quality reads, adapters, contaminant DNA, or PCR duplicates, were removed using Trimmomatic 0.32 (The USADel Lab, Aachen, Germany). Clean data were used for downstream analysis. GC content (%), Q20 (%), and Q30 (%) of the clean data are provided in Supplementary Material Figure S1. De novo reconstruction of transcriptomes from RNA-seq data was performed using Trinity r20410717 (Broad Institute, Cambridge, MA, USA; Herbrew University of Jerusalem, Jerusalem, Israel), which assembles short reads into longer fragments or genes. Bowtie was used for gene alignment and estimation of abundances. A matrix of read counts was created using HTSeq (European Molecular Biology Laboratory, Heidelberg, Germany), which subsequently served as a proxy of transcriptome profiling for each sample.

### 2.4. Identification of Differentially Expressed Genes

Gene expression profiles were compared following the experimental scheme shown in Supplemental Material Figure S1 to determine which genes could be involved in facilitating the thermal tolerance of *H. fuscata* individuals in warmer urban areas. We employed two analysis tools: DESeq2 (European Molecular Biology Laboratory, Heidelberg, Germany) [47] and edgeR (Garvan Institute of Medical Research, New South Wales, Australia; The Walter and Eliza Hall Institute of Medical Research, Victoria, Australia) [48,49] to analyze the differently expressed genes (DEGs). In each analysis, if a contig contained at least one 0 value, it was removed from the analysis. The filtered raw count was log2-transformed and normalized using the run-length encoding method for DESeq2 analysis and trimmed using the M of means method for edgeR analysis. The magnitude of differences in gene expression was examined by determining the fold change (fc). DEGs were defined as those transcripts with |fc| > 2 and an adjusted *p*-value < 0.05. DEGs detected by both DESeq2 and edgeR were employed in gene expression comparisons.

### 2.5. BLAST and Gene Ontology Annotations

BLASTX (https://blast.ncbi.nlm.nih.gov/, accessed on January 2020) was used to annotate each contig of the assemblies against the following protein sequence databases: Gene Ontology Consortium, NCBI non-redundant protein, UniProt, and EggNOG using an E-value default cutoff of 1.0 × 10^−5^. Furthermore, gene ontology enrichment was performed for all contigs using the DAVID tool (https://david.ncifcrf.gov/, accessed on February 2020).

## 3. Results

### 3.1. The Heat Stress Experiment

Three groups of cicadas were examined: the control or unheated group consisted of those cicadas that were not exposed to any heat stress; meanwhile, the 10 min and heat torpor groups refer to those exposed to 10 min of acute heat stress and experienced the heat torpor condition, respectively. The results of heat torpor experiments are provided in Supplementary Material Table S1. After 10 min of acute heat stress, the body temperature of the high-UHI cicadas was 44.93 ± 1.9 °C (*n* = 3, mean ± SD), which was similar to that of the cicadas from the low-UHI area (44.37 ± 4.0 °C, *n* = 3) and the suburban area (43.1 ± 0.6 °C, *n* = 3) (independent samples median Kruskal–Wallis test, χ^2^(2) = 3.60, *p* = 0.165). Nevertheless, high-UHI cicadas became heat torpid at the temperature of 47.77 ± 1.5 °C, which was significantly higher than either the low-UHI (42.37 ± 1.2 °C) or suburban cicadas (44.03 ± 3.6 °C) (independent samples median Kruskal–Wallis test, χ^2^(2) = 6.30, *p* = 0.043). Therefore, the low-UHI and suburban cicadas were expected to exhibit similar gene expressions during both the 10 min and heat torpor experiments.

### 3.2. Illumina Sequencing, De Novo Assembly, and RNA-Seq Mapping

**Figure 1:**
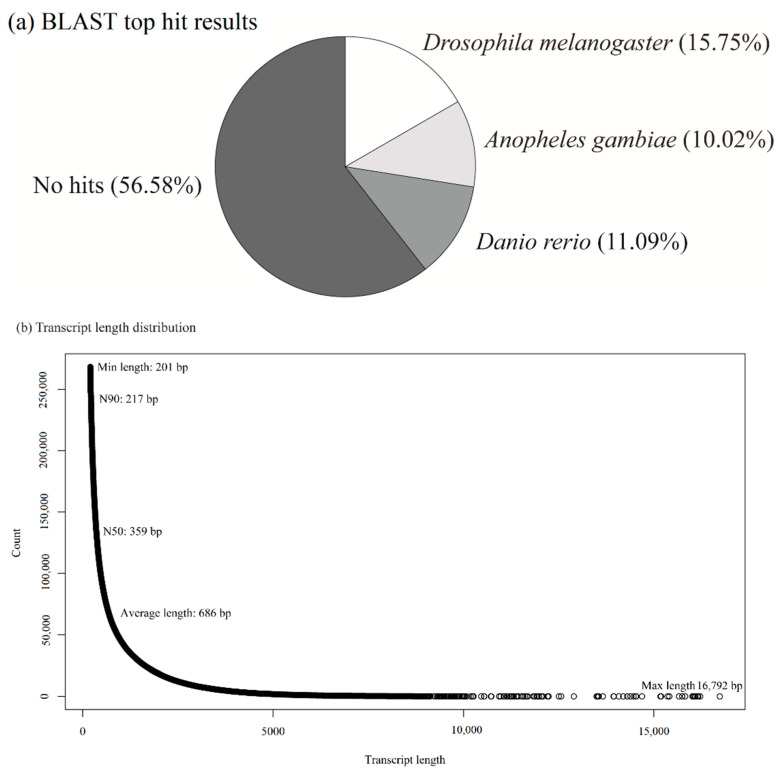
(**a**) BLAST top hit results and (**b**) transcript length distribution. N90: the minimum contig length to which the cumulative length from the largest contig covered 90 percent of the genome; N50: the minimum contig length to which the cumulative length from the largest contig covered 50 percent of the genome; bp: base pairs.

**Figure 2:**
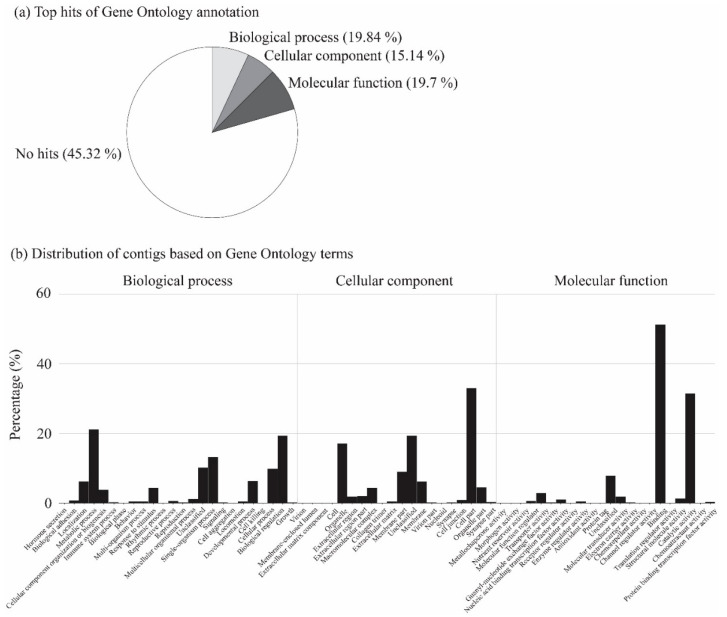
Classification of contigs derived from BLASTX. (**a**) Top hits of the Gene Ontology annotation showed that biological process, cellular component, and molecular function were the three top BLAST hits. The percentage of each category compared with the total number of contigs is provided in brackets. (**b**) Distribution of contigs based on the Gene Ontology terms provides a detailed percentage of the contigs annotated to each mechanism.

RNA-Seq was performed to quantify the variations in gene expressions in relation to heat tolerance in cicadas across the three areas. In total, 27 transcriptome libraries were constructed, where 61 to 83 million clean reads per library were obtained (Supplementary Material Table S1). A pooled de novo transcriptome assembly yielded a total of 261,153 contigs, where 20,201 contigs that did not contain any zero values were employed for further analysis. After annotation using available functional databases, a total of 8772 (43.42%), 11,235 (55.62%), and 8053 (39.86%) contigs were mapped to the Gene Ontology Consortium, NR, and Uniprot databases, respectively. The BLAST taxonomic results of top hits and transcript length distribution are shown in Figure 1. The Gene Ontology term classification revealed that biological process (19.84%), cellular component (15.14%), and molecular function (19.7%) were the top annotation hits (Figure 2). RNA sequence data were submitted to the NCBI Short Read Archive under BioProject accession PRJNA505933.

**Figure 3:**
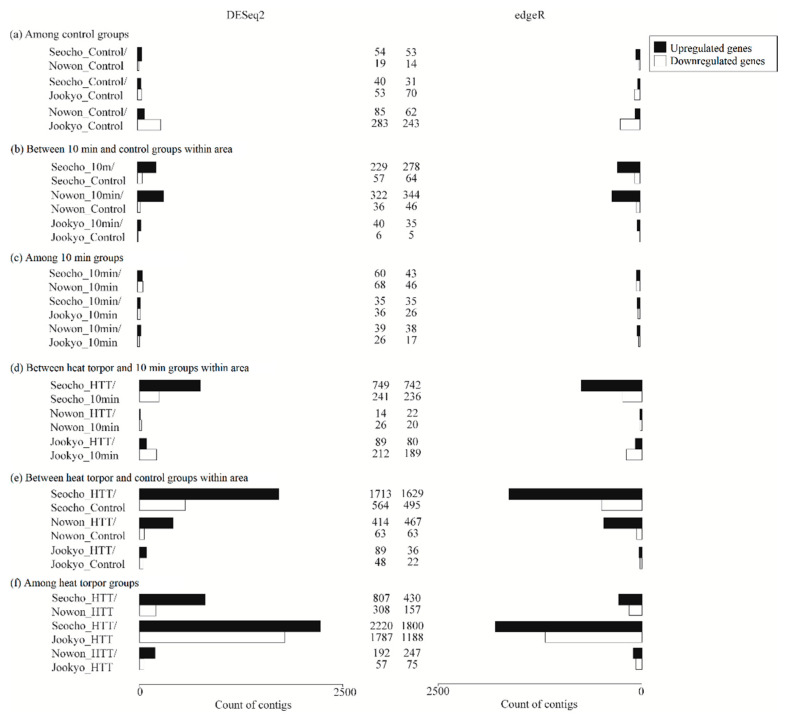
Bar charts presenting the number of significantly differently expressed genes (DEGs) from pairwise comparisons identified using DESeq2 and edgeR analysis platforms. Significantly differently expressed genes satisfied |fc| > 2 and adjusted *p*-values < 0.05. Black bars denote upregulated genes and white bars denote downregulated genes. Seocho, Nowon, and Jookyo represent high-UHI, low-UHI, and surrounding areas, respectively. (**a**–**f**): Pairwise comparisons of number of DEGs of *H. fuscata* cicadas among control groups, between 10 min and control group within area, among 10 min groups, between heat torpor and 10 min groups within area, between heat torpor and control groups within area, and among heat torpor groups, respectively.

In general, the DEGs that were detected using DESeq2 and edgeR were comparable (Figure 3). Heat-torpid cicadas from the high-UHI area expressed remarkably more DEGs than the other groups, and more upregulated than downregulated genes were detected. In particular, most annotated DEGs were metabolic genes, followed by antioxidant and detoxifying genes. These genes are likely to play key roles in facilitating thermal tolerance in cicadas from warm urban areas.

### 3.3. Responses at Normal Conditions

#### Among Control Groups

Unheated samples exhibited a moderately analogous expression pattern in the number of DEGs across the control groups of the three areas (Figure 3a), as well as in the six biological functions (Table 1). Notably, the unheated suburban group showed significantly upregulated expression of one major heat shock 70 kDa protein Ba compared with the unheated high-UHI group, but no Hsps were significantly differentially expressed in other pairwise comparisons.

### 3.4. Acute Heat Stress Responses

After exposure to heat stress for 10 min, a high number of upregulated DEGs were exhibited in cicadas from areas inside Seoul but not in the suburban counterparts (Figure 3b). Nevertheless, differences between the 10 min groups were minor (Figure 3c).

#### 3.4.1. Between 10 min and Control Groups from the Same Area

High- and low-UHI cicadas expressed a comparable amount of DEGs in response to acute heat exposure (Table 1, Figure 3b), whereas only a few genes were differentially expressed in suburban cicadas in response to the acute heat treatment. Except for one downregulated Hsp83 that was detected within high-UHI cicadas, genes involved in metabolism or antioxidants and detoxification showed significantly upregulated responses. The low-UHI cicadas that were exposed to acute heat stress had more upregulated genes related to carbohydrate metabolism than the high-UHI cicadas (29 and 17 DEGs, respectively), in addition to more genes that were annotated as amylase distal (Amy-d), amylase proximal (Amy-p), and amyrel (21 compared with 10 genes in total, respectively). Amy-d, Amy-p, and Amyrel belong to the Amy gene family, whose main function is to degrade starch and other polysaccharides into sugar components and ultimately provide energy using water and oxygen [50,51]. The 10 min high-UHI cicadas expressed a few more DEGs than those from the low-UHI area, and both groups showed upregulation of apolipoprotein D (APOD), cytochrome P450s (CYPs), Neural Lazarillo (NLaz), and lipocalin proteins. APOD and NLaz belong to the lipocalin family, which is an antioxidant protein family whose members increase resistance to heat stress in animals [52,53,54,55]. CYPs are detoxification-related genes that are commonly expressed in heat-stressed organisms [10,56]. Such findings indicate that acute heat stress could trigger transcriptional responses in both high- and low-UHI cicadas.

#### 3.4.2. Among 10 min Groups

Despite the small number of DEGs detected among the 10 min groups, cicadas from both low-UHI and suburban areas showed greater upregulation of heat shock proteins than those from the high-UHI area (Table 1, Figure 3c). In particular, individuals from the low-UHI area expressed seven Hsps at high levels compared with high-UHI cicadas, namely one Hsp60A, two Hsp68s, one Hsp70, one Hsp83, and two heat shock protein 70 cognates (HSC70). Suburban cicadas showed an increase in the expression of five Hsps compared with high-UHI individuals: two Hsp68s, one Hsp83, and two Hsp70s. These results suggested that individuals from low-UHI and suburban areas were more heat-sensitive and a higher degree of heat stress may have built up in those cicadas compared with high-UHI ones.

### 3.5. Heat Torpor Responses

Heat stress in the form of heat torpor invoked remarkable changes in gene expression in cicadas from all areas, as reflected by an increase in the number of both up- and downregulated DEGs in heat torpor groups compared with 10 min or control groups. Cicadas from the high-UHI area expressed the most DEGs, followed by those from low-UHI and suburban areas. As a result, greater variation was detected between high-UHI and suburban cicadas than all other pairwise comparisons.

#### 3.5.1. Between Heat Torpor and 10 min Groups from the same Area

Compared with the 10 min group, heat-torpid cicadas from the high-UHI area had more upregulated genes, particularly those related to heat shock proteins (Hsps), metabolism, and detoxification (Table 1, Figure 3d). Six upregulated Hsp genes comprised two DnaJ heat shock protein family homologs (DnaJ), one heat shock 70 kDa protein cognate, one Hsp68, one Hsp83, and a contig annotated as an Hsp. The expression of Hsps is a universal transcriptional response of insects in general and cicadas in particular to heat stress (18). Thirty-three contigs that were annotated as being involved in carbohydrate metabolism were upregulated, with Amy gene family members accounting for 20 of these contigs. In addition to the previously identified antioxidant proteins APOD and NLaz, peroxiredoxin-4 (prdx4) and glutathione S transferase S1 (GstS1) were found to be upregulated in the high-UHI group. Peroxiredoxin-4 belongs to the peroxiredoxin antioxidant family that protects animals from heat stress [57,58,59]. Similar to CYPs, GstS1 is a detoxification-related gene that is generally expressed in heat-stressed organisms [60]. However, when cicadas were further heat stressed, almost no major changes were observed in the low-UHI area comparison. Cicadas from the suburban area even showed decreased expression of metabolic and detoxifying genes. These findings strongly suggested that cicadas from the warm urban areas had a thermal-tolerating capability that was mediated by changes in their transcriptional responses to heat stress, whereas such responses were lacking in cicadas from the other two areas.

#### 3.5.2. Between Heat Torpor and Control Groups from the Same Area

Comparison of the numbers of DEGs detected by both DESeq2 and edgeR between heat torpor and control groups varied across the three areas (Table 1). Cicadas from the high-UHI area had 1466 upregulated and 426 downregulated DEGs, followed by cicadas from the low-UHI area with 355 upregulated and 53 downregulated DEGs, and finally, suburban cicadas with 32 upregulated and 19 downregulated DEGs. More DEGs were found using DESeq2 than using edgeR (Figure 3e). We focused on genes that were implicated in six biological functions to further understand the overall biology of the transcriptional responses of cicadas to heat stress, namely, heat shock proteins, antioxidants and detoxification, energy metabolism, intracellular immune response, signal transduction pathways, and programmed cell death. Significant expressions of genes involved in these processes were detected mostly within high-UHI cicadas, followed by low-UHI cicadas, while only a few of these genes were detected in suburban cicadas. Genes related to metabolism, antioxidants and detoxification, signal transduction, and protein turnover were primarily upregulated in the high-UHI samples compared with genes related to metabolism and antioxidation in the low-UHI samples.

Ten heat shock proteins were differentially expressed in the high-UHI samples, while none of these Hsps were upregulated in the low-UHI samples. These included members of the heat shock 70 kDa and DnaJ homolog families. Apart from Hsps, a total of 70 DEGs that were annotated as antioxidant and detoxification genes were identified in the high-UHI samples. These included 16 APOD genes, three CYP genes, two Glial Lazarillo (GLaz) genes, five GstS1 genes, four NLaz genes, one lipocalin protein gene, and one prdx4 gene. GLaz is a member of the lipocalin family, in addition to APOD and NLaz. Low-UHI samples also expressed similar antioxidant and detoxification-related genes, namely, six APOD genes, three CYP genes, one GLaz gene, and one NLaz gene. Meanwhile, suburban cicadas only showed upregulated expression of a single CYP gene. Upon exposure to a heat stimulus, not only did high-UHI cicadas express more antioxidant genes than individuals from cooler areas, but the fold change observed in lipocalin family members was remarkable in the high-UHI group compared with the low-UHI group. These results suggested that Hsps and antioxidant and detoxifying genes were induced in cicadas inhabiting the high-UHI area, and likely contributed to their resistance to heat stress.

Numerous pathways related to energy metabolism were enriched in the high-UHI samples, including genes involved in alcohol, carbohydrate, cyclic adenosine monophosphate (cAMP), glutamine, lipid, and nucleoside metabolism. These metabolic processes can provide homeostatic energy during periods of high cellular expenditure [1,7,41]. Within the high-UHI group, the expression of 64 genes involved in the nine aforementioned processes was significantly altered, and most of these genes were upregulated, except for one downregulated DEG. Within the low-UHI group, 39 DEGs involved in four metabolic processes were detected, and all of these genes were upregulated. Within suburban cicadas, only one upregulated DEG involved in carbohydrate metabolism was identified. Besides metabolic pathways, we also found significantly altered expressions of specific genes, such as Amy-d, Amy-p, Amyrel, maltase B1, and thymidine phosphorylase (TYMP). Maltase B1 participates in carbohydrate metabolism, while thymidine phosphorylase is involved in pyrimidine nucleobase metabolism, and the activities of these enzymes promote energy production in the cell [61,62]. Altogether, energy metabolism genes appear to play a vital role in heat tolerance and may facilitate tolerance to heat stress in cicadas.

Under stressful conditions, the innate immune response is also activated for intracellular defense [10]. We observed a difference in the immune responses of cicadas across areas. Within the high-UHI group, among the 19 immune-related DEGs, most were upregulated (14 DEGs). Meanwhile, only one downregulated DEG was found in low-UHI cicadas and none were found in suburban cicadas. The enrichment of genes involved in the Toll signaling pathway, antigen processing and presentation, and scavenger receptors was also determined in high-UHI cicadas, as these genes may play a key role in revoking intracellular immune signals [10]. In addition, many genes involved in signal transduction were enriched in high-UHI cicadas. These genes included members of the Ras superfamily of small GTPases, such as Rab, Ras, and Rho, as well as genes in the Notch signaling pathway. The Ras superfamily is involved in many processes, including cell proliferation, cell adhesion, and apoptosis [63]. Meanwhile, the Notch signaling pathway is a highly conserved cell signaling system in most multicellular organisms and regulates embryonic development [64]. Similar to immune-related genes, most DEGs related to signaling pathways in high-UHI individuals were upregulated (72 out of 97 DEGs). In contrast, low-UHI cicadas expressed only six upregulated DEGs and suburban cicadas exhibited only one DEG. These results signify the critical role of signal transduction in the heat tolerance of cicadas.

When extracellular stress overwhelms the cellular stress response, DNA damage is irreversible and cellular homeostasis is no longer recoverable, causing the activation of death signaling pathways [65]. We found that more genes coding for cell adhesion molecules or E3 ubiquitin-protein ligases, which regulate DNA repair, were upregulated in stressed cicadas from the high-UHI area than cicadas from the suburban and low-UHI areas. Simultaneously, genes related to protein turnover processes were induced in stressed individuals from the high-UHI environment (42 upregulated DEGs, 13 downregulated DEGs), followed by individuals from the low-UHI environment with three upregulated DEGs and suburban cicadas with only one upregulated DEG. Many of those genes were annotated as programmed cell death genes, genes encoding tyrosine- and serine/threonine-specific protein kinases, and genes involved in MAPK or JNK cascades. Both tyrosine- and serine/threonine-specific protein kinases are activated by various stimuli, such as DNA damage or heat shock; while tyrosine kinases specifically target and bind to Ras proteins, ultimately resulting in a signaling cascade [66], serine/threonine kinases activate two homologous yet distinct cascades, namely, the mitogen-activated protein kinase (MAPK) cascade and the c-Jun N-terminal kinase (JNK) cascade, which eventually lead to apoptosis [67]. Greater expression of such processes in stressed cicadas from the high-UHI environment suggested that, despite the presence of several potential thermal adaptation mechanisms, a heat-torpid state was still harmful to cells and that organisms respond at the cellular level by either accelerating the removal of abnormal proteins or intensifying cell repair compared with stressed individuals from low-UHI and suburban areas.

Additionally, four trehalose-6-phosphate synthase 1 (Tps1) contigs were significantly upregulated in heat-stressed samples compared with unheated counterparts for cicadas from both high- and low-UHI environments. The gene Tps1 catalyzes the synthesis of trehalose, which plays an important role in protecting plant and fungi cells from heat stress [68,69,70].

#### 3.5.3. Among Heat Torpor Groups

The most variation was observed between the heat torpor groups from high-UHI and suburban areas, followed by between high- and low-UHI cicadas, whereas the transcription profiles of heat torpor groups from low-UHI and suburban areas showed the least divergence (Figure 3f). The difference in the number of DEGs belonging to the six main biological functions between the areas agreed to some extent with the general pattern of differences in DEGs in heat-torpid cicadas between high-UHI and suburban areas (Table 1).

Specifically, five Hsps were significantly differentially expressed (three downregulated, two upregulated) between heat-torpid high-UHI and suburban cicadas. In addition, there were numerous genes related to antioxidant and detoxification (64 DEGs) and metabolic processes (99 DEGs) that were expressed at higher levels in heat-torpid high-UHI cicadas than suburban cicadas. In particular, critical detoxifying genes described in the previous section, such as those belonging to the lipocalin family, peroxiredoxin family, and cytochrome genes, as well as genes encoding proteins that are involved in numerous metabolic reactions related to energy production showed increased expression in high-UHI versus suburban cicadas. In contrast, high-UHI cicadas displayed decreased expression of intracellular immune-related genes (19 downregulated immune DEGs) and reduced elimination of proteins (50 downregulated protein turnover DEGs) than suburban cicadas. Again, members of the Ras superfamily, their small GTPase-activating proteins, and Notch signaling pathway members accounted for the vast majority of signal transduction DEGs. Many of these DEGs were annotated as being involved in programmed cell death or as encoding tyrosine- and serine/threonine-protein kinases or members of the MAPK or JNK cascades. Heat-torpid cicadas from the high-UHI area expressed a considerable number of DEGs compared with those from the low-UHI area. Although no DEGs related to heat shock proteins or cell repair were detected, high-UHI cicadas tended to have upregulated expression of genes involved in metabolism and detoxification, whereas low-UHI cicadas showed upregulated expression of signal transduction and apoptosis-related genes. These results suggested that the greater heat tolerance of high-UHI cicadas was likely associated with a higher expression of detoxification and energy-metabolic genes, while cicadas from cooler areas probably expressed genes that augmented intracellular protection as a cellular defense against heat stress.

## 4. Discussion

In summary, our main findings showed that high-UHI *H. fuscata* expressed a remarkably different gene pattern compared with those from cooler areas, where several gene-regulating mechanisms were likely involved in facilitating better thermal tolerance under a heat-torpid condition. This was reflected by the increased expression of genes related to heat shock, detoxification, and energy metabolism in *H. fuscata* inhabiting warmer urban areas. Meanwhile, individuals from low-UHI and suburban areas were more heat-sensitive than high-UHI ones, where they showed elevated expression of genes involved in the immune response in addition to signal transduction and apoptosis-related genes related to the elimination of potentially abnormal proteins to protect them from the harmful effects of heat stress.

Exposure to heat stress rapidly induces a cellular stress response to counteract the potentially detrimental effects of the heat by temporarily enhancing tolerance toward macromolecular damage, while at the same time removing irreversibly damaged cells [41]. These attributes are ubiquitous to all cells that have protein chaperones and heat shock proteins [71]. These proteins are of critical importance in protecting cells from stress-induced damage, as they prevent interactions between structurally aberrant proteins or their accumulation and aid in proper folding and restoration of the native activities of proteins and facilitate the degradation or removal of denatured proteins [12,17,18,72,73]. Among numerous heat shock protein families, members of the Hsp70 family are considered to be the major contributors to temperature tolerance in insects [1]. Both Hsp70 and HSC70 function as molecular chaperones by increasing organismal tolerance to hyperthermia (see Feder and Hofmann [12] for a detailed summary of Hsp70 functions). Upregulation of these proteins in the fruit fly *Drosophila* and other insects, such as the green tea leafhopper *Empoasa onukii* [74], larvae of the flesh fly *Sarcophaga similis* [38], the spotted cut-worm *Agrotis c-nigrum* [19], the endoparasitoid *Pteromalus puparum* [28], and the Asiatic rice borer *Chilo suppressalis* [75], were shown to be correlated with the augmentation of tolerance to heat stress and survival [18]. The heat shock gene DnaJ/Hsp40 is a molecular chaperone that aids in the appropriate folding of proteins by binding to denatured proteins and inhibiting their aggregation [18]. Furthermore, DnaJ acts as a co-chaperone of Hsp70; the binding of DnaJ to Hsp70 substantially increases the ability of Hsp70 to interact with a broader spectrum of proteins than Hsp70 itself [76]. Consequently, DnaJ reinforces protein thermostability and accelerates the recovery of damaged proteins in thermotolerant cells [77,78]. In the marine snail *Chlorostoma funebralis*, higher DnaJ expression was observed in the southern population, which may account for the better heat tolerance of this population than the northern population [7]. Wang et al. [11] reported upregulated DnaJ expression in the silkworm *Bombyx mori* females after a high-temperature treatment. In our study, upregulation of Hsps in response to heat stress was observed in high-UHI *H. fuscata*, and Hsps were also upregulated in heat-torpid cicadas from the high-UHI area compared with those from a suburban area, which may contribute to better thermal response of this species in the high-UHI area.

While the predominant function of Hsps is to maintain protein structure in response to heat stress, the heat shock protein 83 (Hsp83) is constitutively transcribed at high levels under normal conditions in both protozoan and eukaryote cells [79,80]. In Leishmania parasites, heat shock moderately attenuated Hsp83 transcription compared with those cultured under normal conditions; nevertheless, elevated heat shock induced an increase in the synthesis of this protein [79]. These findings suggest that under normal conditions, Hsp83 is degraded, whereas heat shock stabilizes the expression of Hsp83, which may explain why no significant alteration in this gene was observed between the control and heat-torpid cicadas from the high-UHI area; however, both these groups had higher transcript levels of Hsp83 than cicadas from the low-UHI area. Hsp83 belongs to the Hsp90 family, which, on one hand, assists in regulating organismal physiological responses to environmental stressors [81] and, on the other hand, plays pleiotropic roles in other critical life-history traits, for instance, development, maturation [82], and reproduction [83,84,85,86]. Heat shock protein 68 belongs to the Hsp70 family [18] and was reported to function as a molecular chaperone in a similar manner to Hsp70 [87]. Hsp60 is another major heat shock protein family whose ATPase activity increases with temperature [18]. Together, such evidence indicates that the upregulation of these Hsps in mildly heated cicadas in low-UHI and suburb areas may help them endure heat stress.

Although Hsp activity promotes organismal heat endurance, overexpression can have deleterious impacts on subsequent growth and cell division, as well as interfere with cellular functions [16,18]. In *Drosophila melanogaster*, long and intense expression of Hsp70 is harmful; it induces higher larval mortality and slows cell growth [12,88,89]. As a result, at high temperatures, natural selection may act to reduce the Hsp70 response [12,16]. This may explain the significant downregulation of several Hsps between heat-torpid cicadas from high-UHI and suburban areas in our study. It was postulated that the expression of Hsps is initiated only when the stressed organism already employs other behavioral or physiological defenses but is unable to prevent detrimental effects [1,22]. Additionally, due to the complexity of modulating Hsp activities and elevated energy expenditure, the expression of Hsps under normal conditions may not be beneficial but rather injurious. The presence of Hsp70 in cell cultures without any stressful exposure halts growth and prevents cell division [90]. Hence, the significantly greater upregulation of the major heat shock 70 kDa protein Ba in unheated cicadas from the suburban area than unheated high-UHI cicadas implied a disadvantage in suburban cicadas. How Hsp83 is induced under normal conditions in cicadas and Leishmania cells remains to be elucidated. Further research should be carried out to clarify the expression pattern and role of each Hsp in cicadas under thermal stress and normal conditions.

Thermal stress also provokes intracellular surplus generation of ROS, which leads to a breakdown of the equilibrium between ROS and antioxidants and oxidative damage to cells, resulting in mutation and apoptosis [29,30,31,32,91,92]. Increased synthesis of antioxidants is thus necessary to scavenge excess ROS and increase thermal tolerance. Numerous antioxidant enzymes are involved in the antioxidative process. Cytochrome P450s and glutathiones are highly conserved antioxidants that can mitigate the deleterious effects of ROS [10]. Cytochrome P450s play key roles in cellular adaptation to oxidative stress by catalyzing the degradation of superoxide radicals and lipid peroxidation [10,93,94,95], thus detoxifying free oxygen radicals and their byproducts [96,97,98]. Meanwhile, glutathione S transferase counteracts the detrimental effects of lipid peroxidation by catalyzing the reduction of lipid peroxides [93,99,100]. Pupae of the tropical tasar silkworm *Antheraea mylitta* exhibited elevated thermal tolerance as antioxidant levels increased [99]. In addition, the peroxiredoxin family is a large family of antioxidant proteins that ubiquitously detoxify peroxides and ameliorate oxidative injury [57]. The marine snail *Chlorostoma funebralis* minimizes oxidative damage by increasing the expression of peroxiredoxin 6 [7]. Other research showed that peroxiredoxin 6 protects mice against oxidative stress and hyperoxia [101,102]. Peroxiredoxin 4 was upregulated to a significantly greater extent following heat stress in high-UHI cicadas than cicadas from the cooler areas, which indicated that this enzyme may protect against thermally induced oxidative stress. In addition to the peroxiredoxin family, we also discovered another large family that was involved in counteracting the damage inflicted by surplus ROS, namely, the lipocalin family. Apolipoprotein D, Glial Lazarillo, and Neural Lazarillo are members of this family that defend against oxidative stress. Studies in *D. melanogaster* revealed that the expression of these lipocalins is boosted by oxidative stress to increase stress resistance and to promote survival [53,54,55,103]. In Drosophila, the amphioxus *Branchiostoma belcheri* [104], and mice [105], a close correlation between increased resistance to hyperoxia and increased expression of those lipocalins was observed. This suggests that the increase in lipocalin expression following heat stress in cicadas from the high-UHI area compared with unheated individuals or to those in low-UHI and suburban areas may result in greater protection against oxidative stress. Apart from being a molecular chaperone that facilitates the normal folding of proteins, Hsp70 has antioxidant activity and was demonstrated to inhibit apoptosis [106,107]. An increase in the expression of these aforementioned antioxidants may not only circumvent heat-induced excessive ROS generation but improve the heat tolerance of *H. fuscata* to a hot environment.

Another key feature of the cellular stress response is the activation of energy metabolism. Under normal conditions, energy expenditure is optimally modulated by cells to provide sufficient energy for basal maintenance (e.g., survival) and additional functions when required (e.g., growth, reproduction) [108]. Upon exposure to heat stress, cells might reallocate their energy budget to prioritize the protection of cells over other energy demands. Genes related to energy metabolism are indispensable to the heat tolerance of an individual, as they encode proteins that provide the necessary energy required for Hsp activities. For Hsps to function effectively, high energy expenditure is required for their synthesis and for salvaging or degrading denatured proteins [18]. Furthermore, apoptosis requires a large portion of the maintenance energy budget in marine bivalves [109,110] such that higher survival of the mussel Mytilus edulis was observed when the apoptotic rate decreased [111,112]. In our study, numerous genes involved in energy metabolic processes and cellular energy were upregulated in heat-stressed *H. fuscata* from the high-UHI environment. These results suggest that under stressful conditions, cicadas inhabiting warm areas may use energy-related metabolic mechanisms to secure cell survival, while simultaneously increasing thermal tolerance toward heat stress. Moreover, as cells predominantly invest energy in basal maintenance upon exposure to heat stress, while other external activities, such as growth and proliferation, are inhibited, the repression of genes related to cell division, cycling, and growth is expected. This phenomenon was observed in several organisms, including the yeast *Saccharomyces cerevisiae* [113], the Iberian freshwater fish *Squalius torgalensis* [8], and the fish *Gillichthys mirabilis* [114]. In our study, cicadas inhabiting a warm urban area showed a much higher expression of genes involved in signal transduction pathways in response to heat stress than cicadas from a cooler urban area. Additionally, there were many more downregulated genes in the high-UHI and suburban area pairwise comparison than other pairwise comparisons. The pivotal role of signal transduction pathways in the heat tolerance of cicadas deserves further careful examination.

In addition to antioxidative and detoxifying mechanisms, the sensing and induction of innate intracellular immune responses are essential to cells. In our study, several immune signaling pathways were activated in response to thermal stress, for instance, the Toll signaling pathway, Toll-like receptors, antigen-processing and presentation-related proteins, and scavenger receptors. The Toll signaling pathway is involved in the response of *Drosophila* to microbial infection, where Toll-like receptors recognize molecular components that are found in bacteria or viruses, thus evoking defense mechanisms [115]. Antigen processing and presentation are features of the adaptive immune response [116]. Scavenger receptors detect and decrease foreign substances and waste material in cells and are involved in apoptosis [117]. The greater upregulation of cellular immune genes in cicadas from cooler areas compared with those in the warm area suggests that heat stress may have a greater adverse effect on cicadas from cool areas, as they require the expression of immune-defense-related genes. In contrast, in the warm urban habitat, innate immune-response-related genes were expressed in heat-stressed individuals compared with unheated individuals, suggesting an essential role of such physiological mechanisms in providing thermal tolerance.

Much effort has been devoted to elucidating how cells enhance thermal tolerance in response to external heat stress. If initial attempts fail to protect cells from macromolecular damage, programmed cell death is essential to eradicate damaged cells [65]. Similar to energy, cells sustain tissue homeostasis under normal conditions with an equilibrium between net growth rate and net cell death rate [42]. Under minor or moderate stress, cellular stress defenses may be sufficient to prevent detrimental stress impacts. In the event of severe stress, cells are no longer able to effectively modulate defense mechanisms, leading to the activation of stress-signaling cascades and eventually cell death pathways. The surplus generation of ROS as a byproduct of heat stress is considered to be a major cause of cell apoptosis [118]. Evidence of heat-induced cell apoptosis was found in the fat bodies of *D. melanogaster* [14] and *Sarcophaga crassipalpis* [119]. In cells, serine/threonine-specific protein kinases regulate several development functions, such as cell proliferation, cell differentiation, embryo development, and programmed cell death [66]. These protein kinases include mitogen-activated protein kinases and stress-activated protein kinase c-Jun N-terminal kinases, whose activation can result in apoptosis [120]. Other protein kinase genes that were differentially expressed in our study were tyrosine-specific protein kinases; the proteins encoded by these genes mainly target Ras protein signal transduction, although they were found to be involved in various signaling cascades [66]. The increased expression of apoptosis-related genes in cicadas from cooler areas compared with those in the warm area suggested less cellular damage to cicadas from the warm area, which further supported their higher thermal tolerance to heat stress. Simultaneously, a large number of protein turnover contigs were expressed in the heat-stressed cicadas than the normal cicadas, indicating that heat-torpid conditions may have been disadvantageous rather than advantageous to cicadas in a warm habitat despite their thermal endurance.

We also identified other genes that appeared to contribute to the enhanced thermotolerance of cicadas, either directly by protecting proteins and membranes from heat stress or indirectly by assisting the activities of other proteins. Trehalose-6-phosphate synthase 1 (TPS1) protects proteins and membranes directly and was widely studied in fungi [121]. The disruption of TPS1 in mutant cells of the yeast Candida albicans and Aspergillus fumigatus impaired their ability to cope with heat stress [68]. However, in the yeasts *Schizosaccharomyces pombe* and *S. cerevisiae*, the overexpression of TPS1 enhanced their tolerance to heat stress [18,122]. Increased expression of TPS1 may therefore benefit *H. fuscata* individuals in high-UHI environments in a similar manner to that observed for yeast.

Upon exposure to heat stress, heat shock genes are instantly activated to defend against potential detrimental stress-induced impairments, maintain proper protein folding, and remove aberrant proteins. Their chaperone function is costly in terms of energy expenditure. At the same time, basal homeostasis for survival also takes precedence over other physiological activities under stressful conditions, such as heat stress. As a result, those individuals who cannot cope with thermal stress may experience irreversible cell death, or at least lower fertility. In contrast, the positive association between the localized thermal regime and thermal responses of cicadas inhabiting warm areas in metropolitan Seoul showed that those individuals could increase their heat endurance as the temperature conditions of their habitat increased [45]. Furthermore, bigger body sizes, which is an indicator of higher reproducibility, were also observed in females living in warm areas, which indicated a possible close relationship between female fecundity and thermal tolerance. We examined the reproducibility of female *H. fuscata* from different areas in Seoul ranging from low to high ambient temperature, and preliminary results showed that those individuals of bigger sizes also had higher fertility (unpublished manuscript). Further detailed analyses are necessary for a comprehensive understanding of how thermal tolerance facilitates better female fecundity.

Overall, we have provided evidence supporting our hypothesis that high-UHI cicadas expressed outstanding transcriptomic profiling compared with those from cooler areas, where several gene-regulating mechanisms were probably involved in facilitating better thermal tolerance under exposure to heat stress. We also showed that heat shock, energy metabolism, and antioxidant genes were expressed to a greater extent in high-UHI cicadas, whereas intracellular immune responses, signal transduction pathways, and genes related to programmed cell death were expressed at greater levels in cicadas from cooler environments. Nevertheless, the distinction between the particular genes that are responsible for better thermal tolerance of high-UHI cicadas compared with their counterparts from cooler areas remains unclear.

## 5. Conclusions

The upregulation of heat shock proteins and increased expression of antioxidant enzymes and energy-metabolism genes were highly expressed in heat-stressed *H. fuscata* cicadas inhabiting high-UHI areas in metropolitan Seoul compared with those in low-UHI and suburban areas. Cicadas with less heat tolerance might be more sensitive to the detrimental impacts of heat stress and probably further upregulated intracellular immune defenses, expended more energy on a protective response against cellular stress, and devoted more energy to remove damaged cells than cicadas with better heat tolerance. Better heat tolerance is advantageous for cicada populations, as adults can utilize more niches that are generated by high UHI conditions, which, in turn, meet the high thermal requirements of the post-morphogenesis of cicada eggs [123], resulting in escalation of the development and growth rate of cicada nymphs underground [124]. This would gradually improve the insect’s ability to adapt to urban environments and ultimately increase its population abundance. Our results support the hypothesis that some gene-regulating mechanisms may facilitate better thermal responses of cicadas in warm urban areas. Nonetheless, more research should be devoted to the clarification of the particular role of each gene in contributing to cicada thermal tolerance.

## Figures and Tables

**Table 1:** Number of differently expressed genes summarized for all transcriptome data and categorized into seven biological functions. Seocho (SC), Nowon (NW), and Jookyo (JK) indicate the high-UHI, low-UHI, and surrounding areas, respectively.

	Total Number of Common Genes		Heat Shock Protein		Cell Repair		Metabolism		Antioxidant and Detoxification		Immune Response		Signal Transduction Pathway		Protein Turnover	
	Up	Down	Up	Down	Up	Down	Up	Down	Up	Down	Up	Down	Up	Down	Up	Down
(a) Among control groups																
SC_control vs. NW_control	46	13	0	0	0	0	3	1	2	2	2	1	0	0	1	0
SC_control vs. JK_control	27	27	0	1	0	0	4	2	0	5	0	2	3	0	1	0
NW_control vs. JK_control	60	192	0	0	0	1	5	6	2	7	1	1	1	2	0	6
(b) Between 10 min and control groups within an area																
SC_10 min vs. SC_control	209	53	0	1	0	0	18	5	12	1	2	1	0	0	1	0
NW_10 min vs. NW_control	291	29	0	0	0	1	38	1	10	0	2	0	6	1	4	0
JK_10 min vs. JK_control	30	4	0	0	0	0	2	0	1	0	0	0	0	0	0	0
(c) Among 10 min groups																
SC_10 min vs. NW_10min	38	42	0	7	0	0	0	0	0	0	2	0	0	0	0	4
SC_10 min vs. JK_10min	28	24	0	5	1	0	0	0	0	0	1	0	0	0	0	1
NW_10 min vs. JK_10min	33	14	0	0	0	0	0	1	0	0	0	0	0	0	2	0
(d) Between heat torpor and 10 min groups within an area																
SC_HTT vs. SC_10 min	683	205	6	0	0	1	48	4	17	2	2	3	5	6	11	2
NW_HTT vs. NW_10 min	12	17	0	0	0	0	2	0	0	0	0	0	1	0	0	0
JK_HTT vs. JK_10 min	54	168	0	1	2	1	5	9	0	5	1	0	0	2	1	1
(e) Between heat torpor and control groups within an area																
SC_HTT vs. SC_control	1466	426	10	0	2	1	128	11	62	8	14	5	72	25	42	13
NW_HTT vs. NW_control	355	53	0	0	1	0	57	1	23	0	1	0	6	0	3	0
JK_HTT vs. JK_control	32	19	0	0	0	0	4	1	1	0	0	0	1	0	1	0
(f) Among heat torpor groups																
SC_HTT vs. NW_HTT	385	116	0	0	0	0	7	6	10	4	2	3	3	13	4	7
SC_HTT vs. JK_HTT	1693	1088	2	3	0	0	99	45	64	18	3	19	17	62	12	50
NW_HTT vs. JK_HTT	168	38	0	0	0	2	7	2	12	0	1	1	1	2	3	0

## Data Availability

The sequencing data produced in this study were deposited in the NCBI Sequence Read Archive under the BioProject accession number PRJNA505933. The accession number for each sample is provided in Supplementary Material Table S1.

## Supplementary Materials

The following are available online at https://www.mdpi.com/article/10.3390/ani11102785/s1, Figure S1: A diagram showing the experimental heat array; Table S1: A summary of transcriptome sequencing data with the accession number of each sample. The Q20 value represents the percentage of bases with a Phred quality score > 20, while Q30 represents a Phred quality score > 30. The body temperatures of the control individuals were not recorded. NA: not available. Seocho, Nowon, and Jookyo represent the high-UHI, low-UHI, and surrounding areas, respectively.
