# Supplementary material for: De Novo Transcriptome Analysis Reveals Potential Thermal Adaptation Mechanisms in the Cicada Hyalessa fuscata
Source: Animals (Basel). 2021 Sep 24;11(10):2785. doi: 10.3390/ani11102785 (PMC8532856; doi:10.3390/ani11102785)
Supplement: Supplementary file 1 [file animals-11-02785-s001.zip › animals-1367127-supplementary.pdf]

Supplementary Material Figure S1. A diagram showing the experimental heat array.

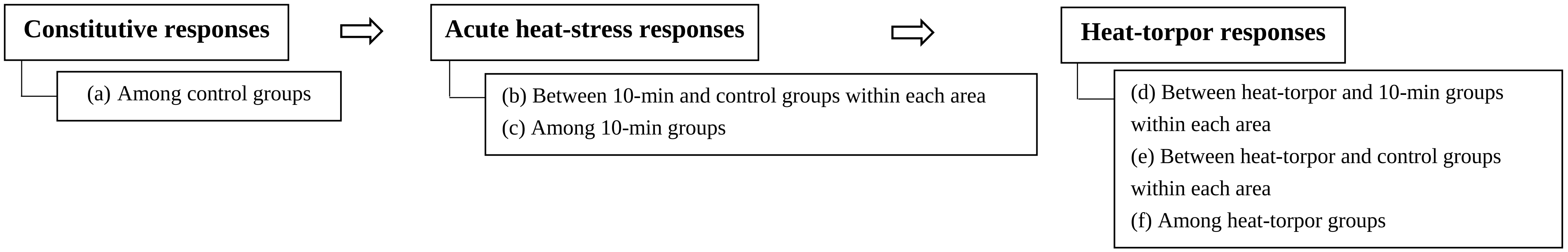

Supplementary Material Table S1. A summary of transcriptome sequencing data with accession number of each sample. Q20 value represents the percentage of bases with a Phred quality score> 20, Q30 a Phred quality score > 30. Body temperatures of controlled individuals were not recorded. NA: not available. Seocho, Nowon and Jookyo represent high-UHI, low-UHI and surrounding areas, respectively.

Sample	Accession number	Examined temperature (ºC)	Raw data				Clean data			
			Read count	GC content (%)	Q20 (%)	Q30 (%)	Read count	GC content (%)	Q20 (%)	Q30 (%)
Seocho_Heat-torpor1	SRR8223459	48.9	63,049,544	43.4	97.62	93.8	61,058,718	43.37	98.61	95.49
Seocho_Heat-torpor2	SRR8223458	48.3	66,491,564	42.76	97.73	94.15	64,575,320	42.73	98.7	95.79
Seocho_Heat-torpor3	SRR8223465	46.1	72,651,622	43.0	97.29	92.61	70,567,074	42.97	98.32	94.36
Seocho_10min1	SRR12603778	45.1	82,218,656	41.01	96.08	91.11	76,975,974	40.98	98.17	94.48
Seocho_10min2	SRR12603777	46.8	83,419,176	41.29	96.69	92.25	79,014,330	41.25	98.40	95.05
Seocho_10min3	SRR12603776	42.9	86,932,330	41.24	97.14	93.10	83,222,432	41.21	98.55	95.40
Seocho_Control1	SRR8223464	NA	68,039,300	40.87	97.61	93.93	66,169,080	40.82	98.64	95.63
Seocho_Control2	SRR8223463	NA	78,195,124	42.03	97.77	94.33	76,216,042	41.96	98.73	95.92
Seocho_Control3	SRR8223462	NA	76,961,634	40.26	97.51	93.73	74,462,202	40.22	98.59	95.56
Nowon_Heat-torpor1	SRR8223457	43.7	67,183,422	42.94	97.7	94.11	65,307,186	42.88	98.69	95.77
Nowon_Heat-torpor2	SRR8223456	41.6	71,937,470	41.22	97.76	94.25	69,897,642	41.17	98.71	95.86
Nowon_Heat-torpor3	SRR8223449	41.8	80,866,470	41.8	97.67	94.12	78,649,532	41.73	98.69	95.81
Nowon_10min1	SRR12603775	40.2	77,189,240	40.90	96.90	92.59	72,208,166	41.25	98.40	95.01
Nowon_10min2	SRR12603774	48.1	86,251,220	41.57	96.98	92.76	73,708,062	41.43	98.30	94.73
Nowon_10min3	SRR12603773	44.8	84,412,456	42.73	97.19	93.13	76,022,904	41.49	98.17	94.41
Nowon_Control1	SRR8223448	NA	82,840,596	41.84	97.87	94.57	80,873,938	41.77	98.79	96.11
Nowon_Control2	SRR8223461	NA	81,135,142	42.0	97.74	94.36	79,047,022	41.92	98.76	96.04
Nowon_Control3	SRR8223460	NA	67,511,472	39.69	98.15	95.15	66,129,196	39.66	98.89	96.39

Jookyo_Heat-torpor1	SRR8223451	46.0	78,470,074	42.71	97.74	94.19	76,499,626	42.65	98.65	95.71
Jookyo_Heat-torpor2	SRR8223450	39.9	79,338,712	41.5	97.6	93.9	77,261,946	41.44	98.58	95.52
Jookyo_Heat-torpor3	SRR8223453	46.2	72,894,416	43.23	97.61	93.97	70,912,696	43.16	98.62	95.64
Jookyo_10min1	SRR12603772	43.4	77,189,240	40.90	96.90	92.59	73,644,456	40.86	98.42	95.09
Jookyo_10min2	SRR12603771	42.4	86,251,220	41.57	96.98	92.76	82,293,132	41.52	98.46	95.18
Jookyo_10min3	SRR12603770	43.5	84,412,456	42.73	97.19	93.13	80,814,960	42.69	98.56	95.40
Jookyo_Control1	SRR8223452	NA	85,213,910	41.19	97.43	93.66	82,659,800	41.12	98.54	95.49
Jookyo_Control2	SRR8223455	NA	69,432,638	40.59	97.6	93.9	67,320,710	40.57	98.58	95.54
Jookyo_Control3	SRR8223454	NA	86,341,120	43.46	97.43	93.48	83,214,332	43.43	98.52	95.33
